# Structural changes of the macula and optic nerve head in the remaining eyes after enucleation for retinoblastoma: an optical coherence tomography study

**DOI:** 10.1186/s12886-017-0650-9

**Published:** 2017-12-16

**Authors:** Azza Mohamed Ahmed Said, Ahmed Mohamed Elbayomi, Ashraf Abdelsalam Kandeel Shaat

**Affiliations:** Ophthalmology Department, Faculty of Medicine, Ain Shams University, Corresponding author address: 10 th Fawzy Elmoteay street, Heliopolis, Cairo Postal code: 11736 Egypt

**Keywords:** Retinoblastoma, Enucleation, Ganglion cell complex, Peripapillary nerve fiber layer

## Abstract

**Background:**

To describe objectively the possible structural changes of the macula and optic nerve head in the free eyes of unilateral cured retinoblastoma patients and, also after enucleation using spectral domain optical coherence tomography.

**Methods:**

A cross sectional study involving 60 patients subdivided into three groups; 15 unilateral RB patients in whom enucleation was indicated as a sole treatment performed earlier in life [(study group (I)], 15 unilateral RB patients who had completely regressed disease with a preserved eye [(study group (II)] and 30 age and sex matched healthy controls. The remaining and free eyes in study groups and right eyes of control group had full ophthalmological examination, static automated perimetry and optical coherence tomography of the macula and optic nerve head.

**Results:**

In study group (II); a significant thinning of total macula, central fovea, ganglion cell layer (GCL), ganglion cell complex (GCC), and some sectors of outer nuclear layer (*P*- values ≤0.05) was found with no significant difference in peripapillary nerve fiber layer (pRNFL) thickness and optic nerve head parameters compared to the control group and the study group (I). A significantly thickened total macula, GCL, GCC, and pRNFL in study group (I) compared to study group (II). Thickened pRNFL was significantly correlated to standard automated perimetry pattern deviations. No significant difference was found between study group (I) and control group.

**Conclusion:**

Retinoblastoma eyes characterized by thinning of central fovea, GCL, GCC compared to the control group. After unilateral enucleation, increased GCC and pRNFL thicknesses were detected compared to retinoblastoma group.

## Background

Retinoblastoma (RB) is the most common type of primary intraocular cancer in children, representing 4% of all pediatric malignancies [1]. It typically presents at a young age, with about two - thirds of all patients diagnosed before 2 years of age [2].

RB1 gene is the first cloned tumor suppressor gene; the protein encoded by this gene is RB protein (RBp) which acts as a cell cycle regulator in the retina and many other tissues [3]. It is a part of a family of proteins including p 103 and p 107, known collectively as ‘pocket proteins’ which induce G1 arrest [4, 5]. RBp influences cell division, survival, and differentiation, but its role in the retina and consequences of its loss on retinal development and differentiation is still unclear and under research. RBp is detectable in the embryonic ganglion cell layer (GCL) but not the primitive retinal neuroepithelium, suggesting that it may regulate cell birth and or differentiation [6, 7]. RB1 inactivation leads to epigenetic changes in key cancer and differentiation pathways in the developing retina [8, 9].

The 5 - year survival rate of more than 95% of all children with RB is the highest of all pediatric cancers in developing countries [1]. Considering the high survival rate of RB patients and the severe impact of the late effects of RB and its treatment, it is important to evaluate health - related quality of life of RB survivors specifically vision related aspects that may result in disabilities, and subsequent restrictions in the activities of daily living which is less well known [10].

Effects of monocular enucleation on ipsilateral and contralateral projections in the higher centers were thoroughly investigated experimentally in different animal species [11–16] long time ago. Objective assessment of the structural changes of the macula and optic nerve head of the remaining free eye after monocular enucleation in human didn’t yet discussed.

New imaging technologies for the early detection and objective evaluation of structural damage of the retina and optic nerve have been developed even before functional damage. Optical coherence tomography (OCT) provides real - time, objective, and reproducible measurements of the optic nerve head and retinal nerve fiber layer [17].

The aim of this study was to describe objectively the possible structural changes of the macula and optic nerve head in the free eyes of unilaterally cured retinoblastoma patients and to compare them with the changes that could be detected in the remaining eyes after enucleation using spectral domain optical coherence tomography.

## Methods

### Study design

A cross sectional study was conducted at Ocular Oncology Clinic, Ophthalmology Department, Ain Shams University in the period from October 2015 to May 2017. The study included 60 eyes of 60 Egyptian patients. Participants were divided into three groups:

- **Study Group (I):** 15 unilateral RB patients in whom enucleation was indicated as a sole treatment performed earlier in life.
- **Study Group (II):** 15 unilateral RB patients who had completely regressed disease with a preserved eye.
- **Control Group:** 30 age and sex matched healthy controls (right eye was tested in each patient).

The clinically free eyes of RB patients were the included eyes in the study. An informed consent was obtained from parents of the study participants. This study was adhered to Declaration of Helsinki Ethical Principles for Medical Research involving Human Subjects and approved by Faculty of Medicine, Ain Shams University Research Ethical Committee (FMASU REC).

Exclusion criteria were; newly diagnosed RB cases and those of less than one year of follow up following complete stoppage of systemic chemotherapy and / or transpupillary diode laser thermotherapy, patients received external beam radiotherapy, brachytherapy and local injections of chemotherapeutic agents as a modality of treatment, Also tumors affecting the macular region (edge of tumor was less than 3 mm from fovea) or were juxtapapillary or associated with localized subretinal fluid or retinal detachment either at time of diagnosis or later on, staging of the disease as Group (C) and (D) at diagnosis according to international intraocular classification of RB [18], also unilateral RB cases with treated tumors of more than 3 mm in base diameter and height and presence of an extraocular disease.

Any participant with media opacities (e.g. corneal opacities, cataract or vitreous hemorrhage), pre-existing retinal or optic nerve diseases either congenial or acquired, spherical equivalent refractive errors of > +/− 3.0 diopters, history of glaucoma, asymmetry of cup/disc (C/D) ratio of ≥0.2 between both eyes in study group (II), unreliable or abnormal standard automated perimetry (SAP) data, previous intraocular surgeries, eye trauma, any systemic diseases (e.g. diabetes mellitus) or neurological disorders (e.g. migraine or demyelinating disease) that may affect accurate and correct interpretation of the results, uncooperative children unable to keep fixation were excluded.

### Ophthalmological examination

All children had full history taken with a special concern about the history of RB. This included age at the time of the present study, sex, age at time of first presentation of RB, duration of follow up, family history, consanguinity, modality of treatment used. No genetic testing was performed to all participants. Ophthalmological examination started by; measurement of best corrected visual acuity (BCVA) using Landolt’s C chart with transformation of it into logarithmic scales according to Log.MAR notation for the purpose of statistical analysis, spherical equivalent calculation, anterior segment examination using slit-lamp biomicroscopy, intraocular pressure (IOP) measurement using Goldmann applanation tonometry and fundus examination by indirect ophthalmoscopy and non contact slit lamp biomicroscopy (Volk 90 D, Volk Optical, Inc., Mentor, USA) for precise examination of the macula and optic disc. Standard automated perimetry (SAP) was performed for cooperative children and spectral domain optical coherence tomography study of all children as follows:

### Standard automated perimetry (SAP)

SAP was performed using a Humphrey Field analyzer 3, model 850 (Zeiss Humphrey Systems, Dublin, CA, USA), with full threshold standard 24–2 algorithm using Goldmann size III white target projected on a 31.8 apostilb (asb) (10 cd per square meter [cd/m^2^]) white background. Reliability criteria were fixation losses of <20% and false positive or false negative response rates <33%. Unreliable data and presence of glaucomatous visual field defects excluded the participant from the study which were detected by two of the following three criteria on SAP: the presence of a cluster of three points on pattern deviation probability plot with a *P*-value of <5%, one of which had a *P*-value <1%, or a pattern standard deviation with a *P*-value <5%, or a glaucoma hemifield test result outside normal limits.

### Optical coherence tomography (OCT) scanning

Optic disc topography, peripapillary retinal nerve fiber layer (pRNFL) thickness and different retinal layers thickness at the macular region were measured using spectral domain OCT machine; Retina Scan RS - 3000 advance (NIDEK Co., Gamagori, Japan) with a scan speed of 53,000 A-scan / second, high quality of images (4 um OCT digital resolution) and real-time, high-contrast and wide view (40° × 30°) of confocal scanning laser ophthalmoscope imaging offers the accuracy for OCT scanning. No normative data base included in the machine for children less than 18 years of age. So a control group was included for comparison.

### Optic nerve head scan

After pupillary dilatation using Tropicamide 1.0% eye drops (Mydriacyl, Alcon Inc., USA), the pRNFL was measured by circular scan around the optic nerve in an area with a diameter of 3.4 mm centered on the optic disc. Overall average pRNFL thickness was automatically calculated along the entire circle. Thickness in each of the superior, nasal, inferior, and temporal quadrants, disc area, cup area and horizontal and vertical cup / disc ratios was measured and compared using disc map over a 6 mm × 6 mm wide area centered on the optic disc. Retinal pigment epithelium tips were detected by the embedded software (NAVIS-EX Image Filing software, RS3000- OCT, NIDEK, Gamagori, Japan), which were refined manually by the operator to delineate the disc and cup margins then the software protocol calculated various parameters that describe the optic nerve head.

### Macula scan

Total Macular thickness was obtained from the macular map scan over a 9 mm × 9 mm wide area image centered on the fovea and calculated automatically by the device. All measurements were evaluated in nine sectors using the Early Treatment Diabetic Retinopathy Study (ETDRS) grid comprising three concentric circles with diameters of 1, 3, and 6 mm. The quadrants were named around the central 1 mm zone [(central foveal thickness (CFT)]; inner superior – nasal – inferior – temporal, and exterior superior – nasal – inferior – temporal from innermost to outermost. Each area was compared with the corresponding area in the control groups.

**Fig. 1:**
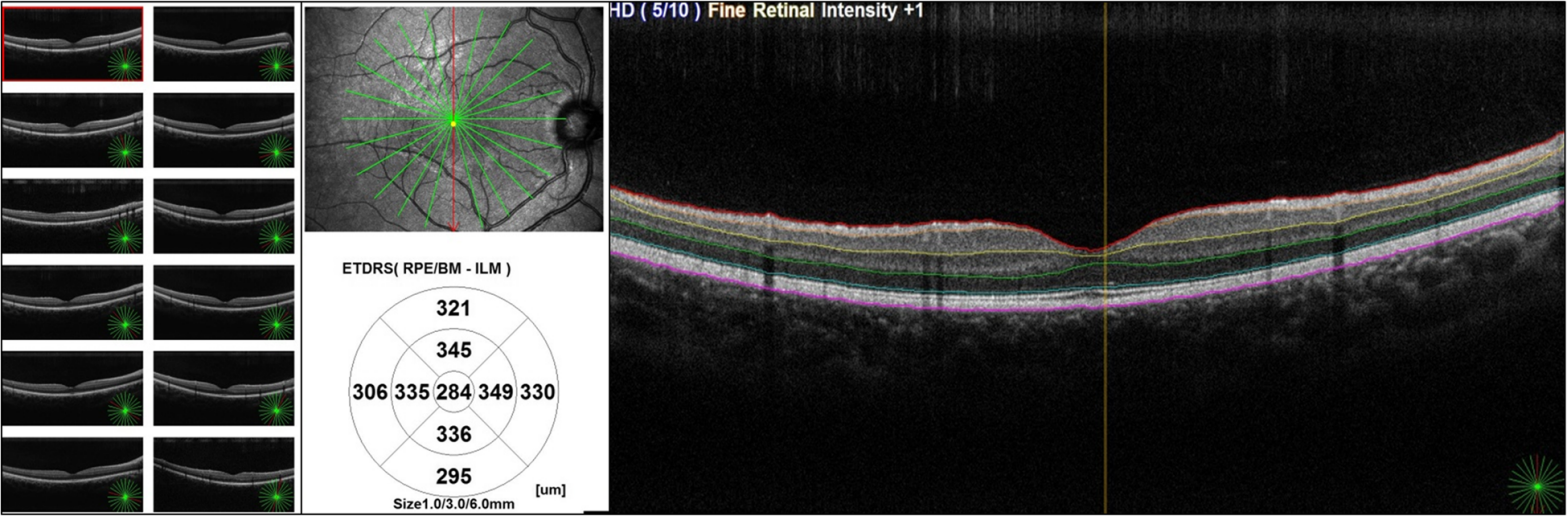
Optical coherence tomography scan of the right macula with 12 radial lines on the left side of the figure. Total macular thickness is automatically calculated in 9 sectors using the ETDRS grid, represented in the middle part. Colored lines on the right part of the figure demonstrated the junctions between various retinal layers

Retinal pigment epithelium to /inner/outer segment junction of photoreceptors (RPE-IS/OS), outer nuclear layer (ONL), Inner nuclear layer (INL), ganglion cell complex (GCC) and macular RNFL (mRNFL) thicknesses were obtained from radial macular scan (Fig. 1). In this scan, 12 lines each 9 mm in length centered on fovea in which the single scan was averaged from 10 A - scans to get the best quality image and reduced noise. The resolution of the single scan of the 10 A - scans was 1.024 points.

The inner nuclear layer (INL) lies between junction of the outer plexiform layer (OPL) / outer nuclear layer (ONL) and inner plexiform layer (IPL) /inner nuclear layer (INL). The macular ganglion cell complex (mGCC) thickness measurement incorporates several retinal layers, including macular ganglion cell layer (mGCL), IPL and overlying mRNFL. Each layer measured also separately, mGCL and mRNFL.

**Fig. 2:**
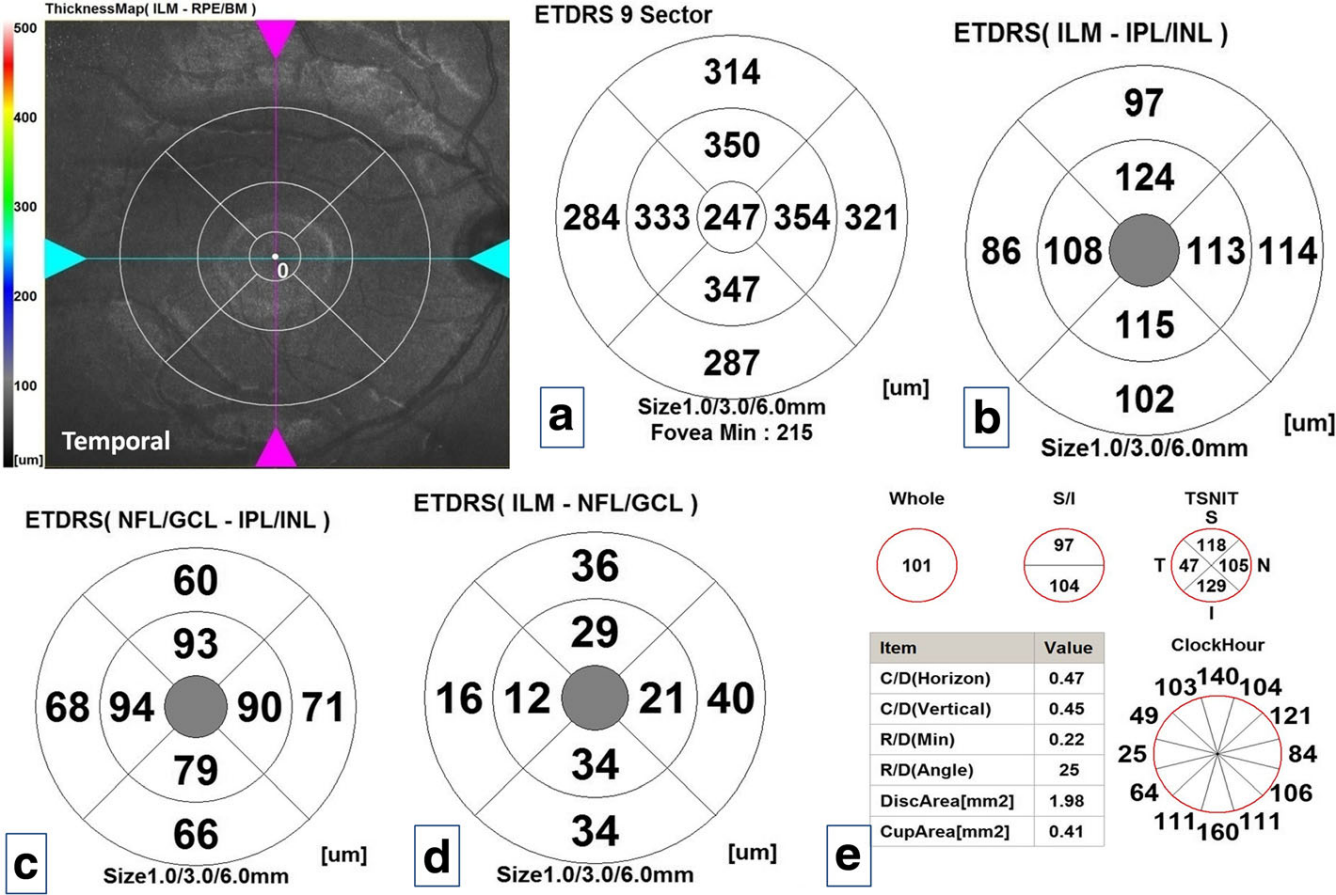
Composite figure of optical coherence tomography maps of the right eye of one subject of the control group. **a** Total macular thickness map. **b** Ganglion cell complex thickness. **c** Macular retinal nerve fiber layer thickness. **d** Ganglion cell layer thickness. **e** Peripapillary retinal nerve fiber layer thickness and optic nerve head parameters

All measurements were evaluated in nine quadrants using the ETDRS grid comprising three concentric circles with diameters of 1, 3, and 6 mm. The quadrants measured were as in total retinal thickness at the macula except the central 1 mm zone which is devoid from GCL, GCC and mRNFL. Figure 2a–e is a composite figure demonstrated different retinal layers thicknesses in the macula and pRNFL and optic nerve head parameters in one subject of the control group.

Patients were instructed to fixate on the intrinsic fixation target during the whole process of OCT scanning. All scans were performed by the same experienced operator (one of the authors). Scans with motion artifacts, segmentation errors, and images with signal strength index less than 45 dB were excluded.

### Statistical analysis

All data were analyzed using SPSS 13 software (SPSS Inc., Chicago, Illinois, USA). Kolmogorov–Smirnov test was used to assess normality of data. Parametric continuous variables were expressed as mean and standard deviation (SD). Independent sample t- test was used to compare between quantitative variables of two groups. One – way Analysis of variance (Anova) test was used to compare between variables of more than two groups. Description of qualitative variables was in the form of numbers and percentages. Chi - square test was used to compare between qualitative data. Non - parametric quantitative data was expressed as median and interquartile range (IQR), 95% confidence interval for mean. Comparison between variables of independent samples was performed using Mann – Whitney test. For comparison between more than two groups, Kruskal-Wallis test was used. Pearson’s correlation coefficient was used to assess the correlation between different variables. The level of significance was set at *P*-value ≤0.05.

## Results

The study included 41 (68.3%) males and 19 (31.7%) females with a mean age of 11.95 years ±4.0 SD (median: 12, range: 5–19). Mean BCVA (Log.MAR) in the studied eyes was 0.1 ± 0.03 SD (median: 0.1, range: 0.1–0.3) and mean spherical equivalent of 1.0 diopters ±0.73 SD (median: 1.0, range: −1.25 to +2.75). Mean IOP was 11.67 mmHg ±1.7 SD (median 12, range: 9–15). Average SAP mean deviation of −1.44 dB ± 1.48 SD (median: −1.19, range: - 2.01 to – 4.29) and pattern standard deviation of 1.14 dB ± 1.03 SD (median: 1.14, range: 0.0–3.1). The mean age at time of presentation of RB was 23.13 months ±13.36 SD (median: 24, range: 2.0–48) and 15.20 months ±7.76 SD (median: 15, range: 5.0–26.0) in study groups (I) and (II) respectively. The mean duration of follow up of RB children was 7.93 years ±3.49 SD (median: 7.5, range: 3.5–17) and 10.38 years ±3.73 SD (median: 8.4, range: 7.0–17.5) in study groups (I) and (II) respectively. There was no statistically significant difference between the control and study groups regarding all demographic data (*P*- values were >0.05). Table 1 summarized demographic data of the control group and study groups (I) and (II).

**Table 1:** Comparison between the control group and study groups as regards demographic data using independent sample *t*-test

Parameter	Control group	Study group (I)	Study group (II)	*P*-value [control group versus study group (I)]	*P*-value [control group versus study group (II)]	*P*-value [study group (I) versus study group (II)]
	Mean ± SD					
Mean age (years)	12.77 ± 4.16	10.73 ± 4.0	11.53 ± 3.49	0.12	0.30	0.56
Mean age at presentation (months)	N/A	23.13 ± 13.36	15.20 ± 7.76	N/A	N/A	0.06
Sex distribution No. (%)						
Male	24 (80.0)	8 (53.3)	9 (60.0)	0.06	0.15	0.71^a^
Female	6 (20.0)	7 (46.7)	6 (40.0)			
Diagnosis of retinoblastoma No. (%)						
Right	N/A	7 (46.7)	10 (66.7)	N/A	N/A	0.27^a^
Left		8 (53.3)	5 (33.3)			
Family history No. (%)						
Negative	N/A	15 (100.0)	14 (93.3)	N/A	N/A	1.0^a^
Positive		0 (0.0)	1 (6.7)			
Consanguinity No. (%)						
Negative	N/A	13 (86.7)	15 (100.0)	N/A	N/A	0.48^a^
Positive		2 (13.3)	0 (0.0)			
Mean duration of follow up (years)	N/A	7.93 ± 3.49	10.38 ± 3.73	N/A	N/A	0.07
Mean BCVA (Log.MAR)	0.11 ± 0.03	0.11 ± 0.05	0.11 ± 0.02	0.64	1.00	0.66
Mean spherical Equivalent (diopters)	0.83 ± 0.72	1.28 ± 0.82	1.12 ± 0.60	0.08	0.16	0.53
Mean IOP (mmHg)	11.6 ± 1.69	11.9 ± 1.87	11.53 ± 1.64	0.56	0.90	0.54
Average of mean deviation (decibels)	−1.37 ± 1.43	−1.62 ± 1.29	−1.41 ± 1.80	0.56	0.93	0.72
Mean of pattern standard deviation (decibels)	1.14 ± 1.05	1.12 ± 1.04	1.14 ± 1.07	0.95	0.99	0.95

*N/A*, not applicable; *SD*, standard deviation; *No*., number; *Log.MAR*, logarithm of minimum angle of resolution

^a^Comparison by Chi-square test

A statistically significant difference was found between the three groups regarding the mean CFT using one - way Anova test (*P*- value = 0.008). It was statistically significantly lower in study group (I) and study group (II) compared to the control group.

Regarding the mean total macular thickness map; a statistically significant difference between the three groups regarding superior sectors in the inner 3 mm ring, outer 6 mm ring, and the temporal sector in 6 mm ring (*P*-values were 0.031, 0.30 and 0.029 respectively). A statistically significant thinner macular thickness in all sectors in study group (II) compared to each of the control group and study group (I) except in the inferior sectors.

Table 2 demonstrated the comparison between the control and study groups regarding total and inner retinal layers thicknesses at the macula.

**Table 2:** Comparison between the control group and study groups as regards total and inner retinal layers thicknesses at the macula using independent sample *t*-test

Parameter	Control group	Study group (I)	Study group (II)	*P*-value [control group versus study group (I)]	*P*-value [control group versus study group (II)]	*P*-value [study group (I) versus study group (II)]
	Mean (μm) ± SD					
A. *Central foveal thickness (Central 1 mm)*	268.09 ± 23.49	246.8 ± 15.33	249.85 ± 17.69	0.001	0.04	0.71
B. *Total macular thickness*						
*3 mm ring*						
Superior	350.93 ± 21.87	343.08 ± 12.02	331.42 ± 10.17	0.08	0.002	0.04
Nasal	345.13 ± 17.77	342.14 ± 9.29	330.85 ± 10.15	0.12	0.01	0.03
Temporal	330.64 ± 15.26	332.35 ± 12.52	318.28 ± 8.38	0.60	0.009	0.01
Inferior	336.87 ± 21.34	338.83 ± 12.76	330.42 ± 12.25	0.71	0.3	0.18
*6 mm ring*						
Superior	307.29 ± 15.82	310.00 ± 1.15	293.42 ± 8.24	0.43	0.004	0.001
Nasal	322.93 ± 16.64	322.58 ± 12.6	310.14 ± 10.94	0.94	0.026	0.04
Temporal	291.06 ± 14.18	297.42 ± 10.23	280.28 ± 11.44	0.11	0.05	0.007
Inferior	300.64 ± 19.12	303.91 ± 14.93	292.85 ± 13.51	0.56	0.23	0.12
C. *Mac RNFL thickness*						
*Average Mac RNFL*	27.35 ± 5.02	28.19 ± 4.85	23.55 ± 4.72	0.62	0.08	0.06
*3 mm ring*						
Superior	27.93 ± 7.19	26.5 ± 12.0^a^	22.14 ± 6.01	0.90^b^	0.05	0.08^b^
Nasal	20.16 ± 7.96	19.68 ± 7.35	17.14 ± 5.63	0.92	0.26	0.35
Temporal	12.77 ± 8.61	43.83 ± 7.98	9.42 ± 7.29	0.46	0.31	0.16
Inferior	24.64 ± 6.23	24.08 ± 7.31	20.85 ± 4.48	0.81	0.08	0.25
*6 mm ring*						
Superior	35.48 ± 3.62	37.83 ± 6.60	33.0 ± 5.53	0.26	0.29	0.11
Nasal	45.13 ± 6.72	43.0 ± 2.75^a^	40.57 ± 6.37	0.33^b^	0.12	0.15^b^
Temporal	15.96 ± 6.27	18.08 ± 6.25	12.71 ± 4.99	0.33	0.16	0.06
Inferior	36.74 ± 4.39	36.75 ± 7.27	32.57 ± 4.99	0.99	0.07	0.16
D. *Ganglion cell layer (GCL) thickness*						
*Average GCL thickness*	80.76 ± 6.93	80.04 ± 4.59	74.45 ± 4.49	0.69	0.01	0.02
*3 mm ring*						
Superior	93.32 ± 9.35	91.0 ± 6.03	86.85 ± 5.78	0.16	0.03	0.16
Nasal	97.45 ± 8.67	93.91 ± 6.69	89.14 ± 6.59	0.17	0.02	0.15
Temporal	92.64 ± 13.17	90.58 ± 6.34	80.57 ± 9.50	0.49	0.01	0.03
Inferior	92.87 ± 11.64	90.57 ± 8.34	86.28 ± 8.63	0.48	0.12	0.31
*6 mm ring*						
Superior	61.25 ± 6.32	62.41 ± 6.45	60.0 ± 3.0^a^	0.60	0.51^b^	0.33^b^
Nasal	70.54 ± 6.45	71.33 ± 4.88	64.28 ± 6.89	0.67	0.06	0.04
Temporal	72.64 ± 8.68	74.08 ± 7.85	70.43 ± 4.57	0.61	0.35	0.21
Inferior	65.35 ± 8.72	66.41 ± 6.67	59.28 ± 8.86	0.67	0.13	0.09
E. *Ganglion cell complex (GCC) thickness*						
*Average GCC thickness*	108.10 ± 7.39	108.62 ± 2.64	98.0 ± 6.42	0.87	0.004	0.004
*3 mm ring*						
Superior	121.19 ± 10.37	119.5 ± 8.5^a^	109.0 ± 9.42	0.68^b^	0.01	0.01^b^
Nasal	117.54 ± 8.64	113.83 ± 5.81	106.28 ± 5.15	0.11	0.0004	0.01
Temporal	105.32 ± 9.66	105.58 ± 3.98	90.0 ± 8.98	0.91	0.003	0.003
Inferior	117.61 ± 11.9	116.0 ± 4.31	107.14 ± 9.97	0.39	0.04	0.07
*6 mm ring*						
Superior	96.64 ± 7.96	100.25 ± 6.67	91.71 ± 4.95	0.14	0.05	0.006
Nasal	115.74 ± 9.50	114.91 ± 5.64	104.85 ± 9.65	0.73	0.02	0.034
Temporal	88.61 ± 8.38	92.17 ± 5.50	83.14 ± 3.08	0.11	0.008	< 0.001
Inferior	102.16 ± 10.71	103.17 ± 7.12	91.86 ± 13.25	0.72	0.09	0.07

*SD*, standard deviation; *μm*, micron

^a^Median ± Interquartile range;^b^Comparison by Mann Whitney U test

Comparison between the three groups regarding the mean GCL thicknesses in all quadrants show a statistically significant difference in nasal sectors in inner 3 mm ring, 6 mm ring and the temporal sector in 3 mm ring (*P*-value were 0.048, 0.05 and 0.043 respectively).

The mean GCL was significantly lower in study group (II) compared to each of the control group and study group (I). The superior sector in the 3 mm ring was the most thinner statistically in study group (II) when compared to the control group, and the nasal sector in the 6 mm ring was most thinner statistically in study group (II) when compared to the study group (I).

Comparison between the three groups revealed a statistically significant lower value in the overall GCC in study group (II) (P- value was 0.002). Comparison was done between the three groups regarding the mean GCC in all sectors show a statistically significant difference in nasal and temporal sectors in inner 3 mm ring and 6 mm ring (*P*-values were, 0.004, 0.017, < 0.01 and 0.042 respectively). Also a statistically difference was found in superior sector in 3 mm ring and inferior sector in 6 mm ring (*P*-value was 0.02 using Kruskal-Wallis test and was 0.05 using one – way Anova test respectively).

The mean GCC thickness was statistically significant lower in study group (II) compared to each of the control group and study group (I) with affection of all sectors except the inferior sectors which were statistically preserved.

The mean ONL thickness was preserved in all sectors with no statistically significant difference between the control group and the study groups except in superior, nasal and inferior sectors which were statistically lower in study group (II) compared to control group and study group (I). Table 3 provided the comparison between the control and study groups regarding inner nuclear, outer nuclear and RPE- IS/OS layer thicknesses at the macula.

**Table 3:** Comparison between the control group and study groups as regards inner nuclear layer, outer nuclear layer and RPE-IS/OS complex thicknesses at the macula using independent sample *t*-test

	Control group	Study group (I)	Study group (II)	*P*-value [control group versus study group (I)]	*P*-value [control group versus study group (II)]	*P*-value [study group (I) versus study group (II)
	Mean (μm) ± SD					
F. *Inner nuclear layer thickness*						
*3 mm ring*						
Superior	84.13 ± 9.72	85.25 ± 8.53	80.57 ± 15.76	0.71	0.58	0.49
Nasal	82.55 ± 11.97	82.41 ± 12.33	83.71 ± 12.75	0.97	0.83	0.83
Temporal	76.64 ± 5.43	75.17 ± 7.14	74.85 ± 7.65	0.52	0.57	0.93
Inferior	79.52 ± 9.09	80.17 ± 6.28	74.86 ± 11.24	0.79	0.34	0.28
*6 mm ring*						
Superior	69.87 ± 20.27	65.33 ± 4.67	64.43 ± 7.20	0.25	0.24	0.77
Nasal	69.64 ± 8.04	69.08 ± 5.14	68.15 ± 10.12	0.78	0.72	0.82
Temporal	67.29 ± 4.66	66.0 ± 2.0^a^	64.71 ± 4.15	0.61^b^	0.18	0.61^b^
Inferior	67.54 ± 7.86	66.75 ± 4.81	62.71 ± 9.55	0.69	0.25	0.33
G. *Outer retinal layers thickness*						
• *ONL thickness*						
*1 mm central thickness*	85.38 ± 11.51	82.91 ± 9.93	79.00 ± 6.42	0.49	0.06	0.31
*3 mm ring*						
Superior	66.96 ± 9.06	67.41 ± 8.26	64.57 ± 11.41	0.87	0.62	0.57
Nasal	72.41 ± 9.95	69.08 ± 13.37	62.58 ± 9.46	0.45	0.04	0.23
Temporal	72.06 ± 9.34	73.83 ± 6.22	64.57 ± 10.58	0.47	0.12	0.06
Inferior	67.87 ± 11.28	68.25 ± 12.7	65.85 ± 6.84	0.93	0.54	0.61
*6 mm ring*						
Superior	64.22 ± 7.71	65.25 ± 6.59	58.71 ± 6.34	0.67	0.07	0.05
Nasal	63.51 ± 9.05	66.0 ± 17.75^a^	57.14 ± 5.89	0.90^b^	0.04	0.17^b^
Temporal	64.48 ± 8.57	67.16 ± 5.13	61.28 ± 6.77	0.22	0.31	0.07
Inferior	59.25 ± 9.63	60.08 ± 8.24	54.00 ± 4.86	0.78	0.05	0.05
• *RPE- IS/OS layer*						
*1 mm central thickness*	75.06 ± 2.89	75.0 ± 1.95	74.28 ± 2.43	0.93	0.48	0.52
*3 mm ring*						
Superior	68.52 ± 3.81	67.25 ± 2.59	68.00 ± 4.00	0.22	0.76	0.67
Nasal	69.09 ± 2.99	68.67 ± 2.35	67.71 ± 1.70	0.62	0.12	0.32
Temporal	68.93 ± 3.13	68.67 ± 3.20	68.71 ± 3.25	0.81	0.87	0.97
Inferior	67.93 ± 3.73	68.08 ± 2.81	66.72 ± 1.97	0.89	0.24	0.23
*6 mm ring*						
Superior	68.51 ± 2.58	66.50 ± 2.61	66.28 ± 2.21	0.03	0.04	0.85
Nasal	67.42 ± 2.17	66.58 ± 2.93	65.57 ± 2.37	0.38	0.09	0.42
Temporal	66.19 ± 2.73	65.91 ± 2.50	65.0 ± 4.0^a^	0.75	0.04^b^	0.31^b^
Inferior	66.61 ± 2.17	66.00 ± 2.73	65.71 ± 2.14	0.49	0.34	0.80

SD, standard deviation; μm, micron

^a^Median ± Interquartile range;^b^Comparison by Mann Whitney U test

A statistically significant difference was found in superior sector in 6 mm ring when comparison was done between the three groups using Kruskal-Wallis test. The mean RPE- IS/OS layer was statistically significant thinner in superior and inferior sectors in 6 mm ring in study group (II) compared to control group and study group (I).

There was a statistically significant difference regarding mean pRNFL thickness and the inferior quadrant RNFL thickness among the three groups (*P*-values were 0.003 and 0.017 respectively using Kruskal-Wallis test).

The mean pRNFL thickness was statistically thicker in the study group (I) when compared to control group, All optic nerve head quadrants were statistically significantly thicker except the nasal quadrant. There was a statistically significant thin average pRNFL thickness, thickness of superior, temporal and inferior quadrants in study group (II) compared to study group (I).

Table 4 show comparison between the control and study groups as regards the average pRNFL thickness and the other optic disc parameters.

**Table 4:** Comparison between the control group and study groups as regards pRNFL and optic disc parameters using independent sample *t*-test

	Control group	Study group (I)	Study Group (II)	*P*-value	*P*-value	*P*-value
	Mean (μm) ± SD					
H. *Peripapillary retinal nerve fiber layer (RNFL) thickness*						
Average	105.29 ± 6.64	114.58 ± 7.76	101.28 ± 12.22	0.002	0.43	0.05
Superior	134.64 ± 16.12	152.58 ± 12.0	140.43 ± 19.16	0.0005	0.39	0.04
Nasal	84.64 ± 14.40	84.25 ± 12.24	85.57 ± 24.71	0.93	0.93	0.89
Temporal	65.83 ± 12.31	74.83 ± 7.90	68.57 ± 14.08	0.008	0.73	0.03
Inferior	132.61 ± 16.19	148.83 ± 15.98	125.57 ± 24.09	0.007	0.48	0.05
I. *Optic disc parameters*						
Horizontal C/D ratio	0.37 ± 0.25	0.47 ± 0.12	0.45 ± 0.12	0.09	0.22	0.76
Vertical C/D ratio	0.35 ± 0.23	0.43 ± 0.11	0.41 ± 0.09	0.15	0.36	0.59
Disc area (mm^2^)	2.13 ± 0.52	2.06 ± 0.33	2.28 ± 0.33	0.63	0.34	0.19
Cup area (mm^2^)	0.48 ± 0.46	0.37 ± 0.42^a^	0.45 ± 0.17	0.93^b^	0.75	0.7^b^

SD, standard deviation; μm, micron; C/D, cup disc ratio; mm, millimeter

^a^Median ± Interquartile range;^b^Comparison by Mann Whitney U test

**Fig. 3:**
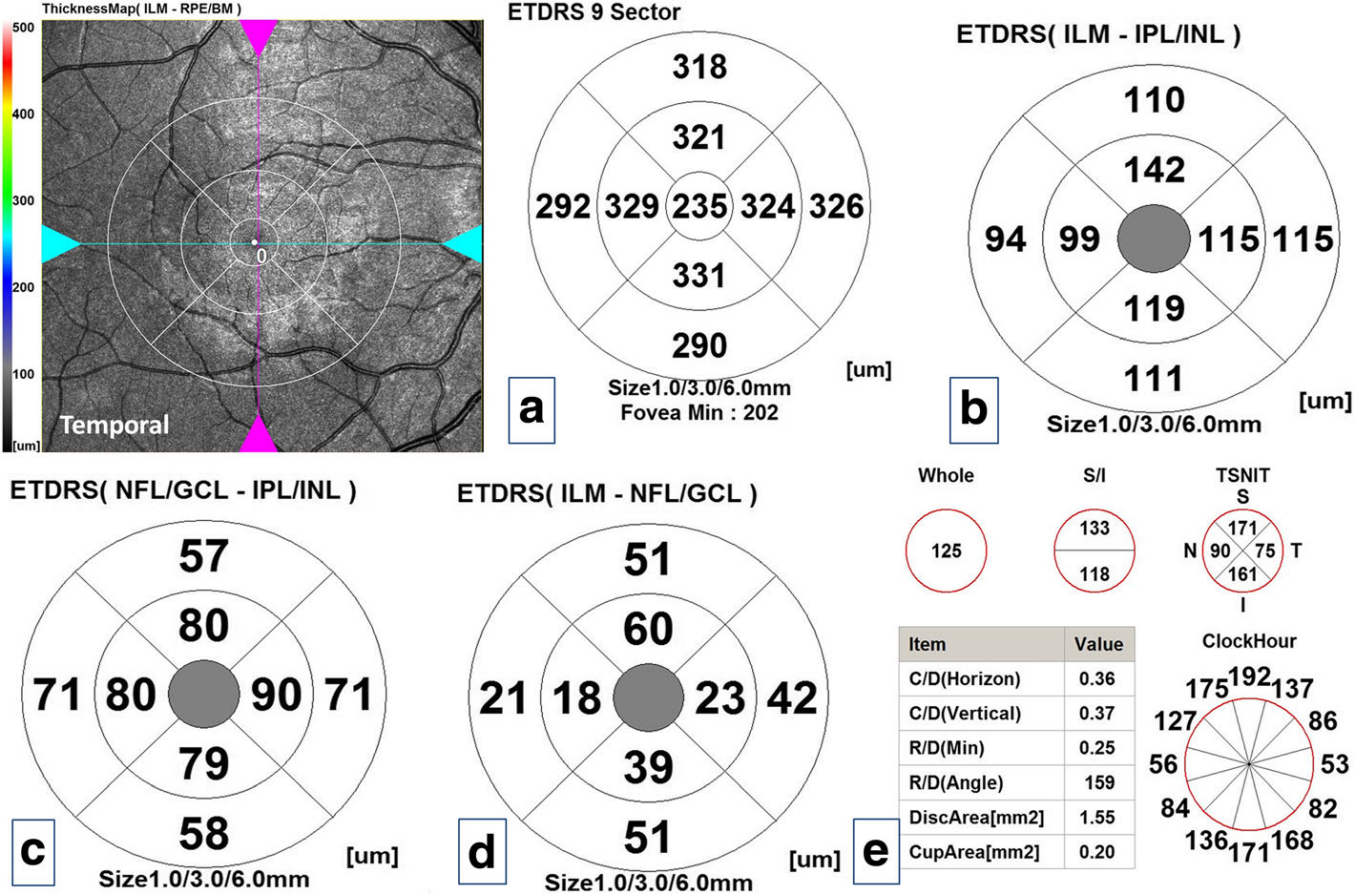
Composite figure of optical coherence tomography maps of the right eye of one of the patients of study group (I) [remaining eye after enucleation]. **a** Total macular thickness map. **b** Ganglion cell complex thickness. **c** Ganglion cell layer thickness. **d** Macular retinal nerve fiber layer thickness. **e** Peripapillary retinal nerve fiber layer thickness and optic nerve head parameters. Notice thickened all maps compared to Fig. 4 [free retinoblastoma eye] except the ganglion cell layer thickness map and almost no differences in all maps when compared to corresponding in control group in Fig. 1 except the peripapillary nerve fiber layer thickness in all sectors which was thickened

**Fig. 4:**
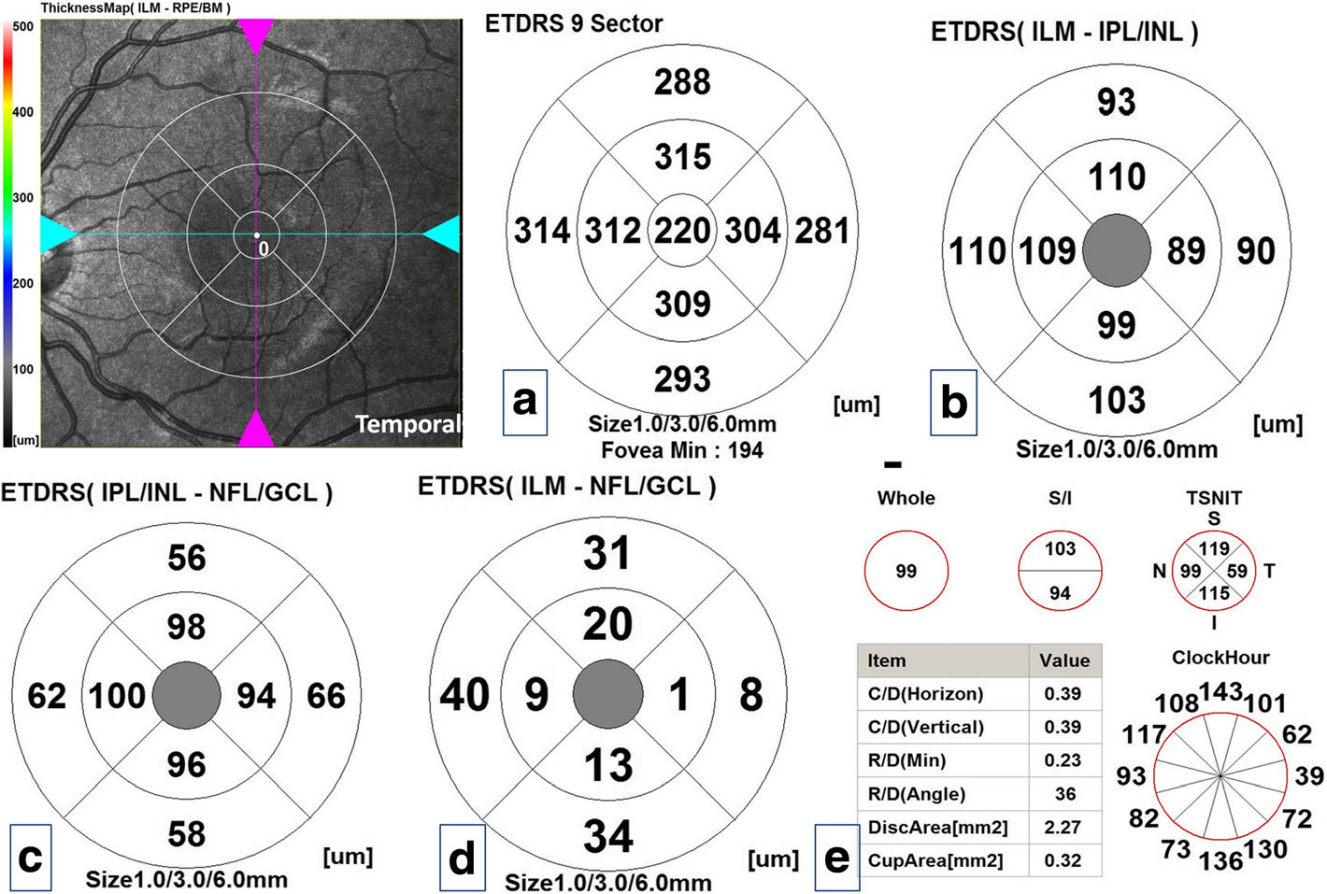
Composite figure of optical coherence tomography maps of the left eye of one of the patients of study group (II) [clinically free eye of retinoblastoma]. **a** Total macular thickness map. **b** Ganglion cell complex thickness. **c** Ganglion cell layer thickness. **d** Macular retinal nerve fiber layer thickness. **e** Peripapillary retinal nerve fiber layer thickness and optic nerve head parameters. Notice thinning of all maps compared to the control group except the corresponding nasal and inferior sectors of total macular thickness, ganglion cell complex and macular retinal nerve fiber layer and notice the concentric thinning of 6 mm ring of ganglion cell layer

Figures 3 and 4a, e demonstrated retinal layers thicknesses at the macula and pRNFL thickness and optic nerve head parameters in one of our patients in study group (I) and (II) respectively. Pearson’s correlation coefficient was done between average macular layers thicknesses and average pRNFL thickness and different variables: mean age, sex, family history, consanguinity, duration of follow up, BCVA, spherical equivalent, IOP, mean SAP mean deviation and pattern standard deviation revealed no statistically significant correlations (*P*-values were >0.05) except a statistically significant positive moderate correlation in the enucleation group [study group (I)] between mean pRNFL thickness and mean age at the time of the study and mean SAP pattern standard deviation (r:0.65, *P*-value was 0.02 and r: 0.67, *P*-value was 0.02 respectively).

## Discussion

In the current study, thinning of retinal layers in the free eyes of the RB survivors was detected compared to that in the control group. This was statistically significant in the mean CFT, GCL, GCC, total macular thicknesses and some sectors of ONL. This was combined with preservation of the mean thicknesses of almost all RPE IS/OS layer, pRNFL and the other optic nerve head parameters. All those RB patients had normal vision according to World Health Organization guidelines [19]. The present study was the first study to report these findings. Indeed, to understand why this thinning occurred we reviewed how the retina developed and the role of RBp during cell cycle, division and differentiation and the effects of its loss on the retinal development beside RB tumor formation in certain locations.

All studies on this context was in animal models, two studies [20, 21] show that in the absence of RBp, the mature retina lacks ganglion and bipolar cells and has fewer rods and in the absence of both RBp and p107; ganglion, bipolar, rod and cone cells are all deleted. However, amacrine, horizontal and Muller cells survive either RBp or RBp and p107 loss.

In addition to the known role of RBp in controlling cell division, it binds and potentiates several transcription factors involved in differentiation [22, 23].

RB - deficient retinas had ectopic S - phase and high levels of p53 - independent apoptosis, especially in the differentiating retinal GCL in late embryogenesis and adults showed loss of photoreceptors and bipolar cells [21].

On the other hand, Vooijs et al. [24] in their study suggested that RBp, p107 and p53 are not required for terminal differentiation of photoreceptors. Continued proliferation of photoreceptor precursors or phenotype plasticity of the neuronal and glial cells reduced the effects of RBp deficiency in the neural retina [25].

Brg1 (Smarca4) is an ATPase subunit and a tumor suppressor in RB, It was found to regulate retinal size by controlling cell cycle length, cell cycle exit and cell survival during development. The combination of defective cell differentiation and lamination led to retinal hypocellularity in all three cellular layers of the retina and degeneration in Brg1-deficient retinae [26]. So RBp and Brg1 defects may be considered as a cause of CFT, GCL, GCC layers thinning in the maculae of free eyes in study group (II).

Cyclins and cyclin dependent kinases are direct regulators of RB family. If persistent expression of cyclin D1 occurred due to genetic defects, it disrupts photoreceptor differentiation as manifested by delayed expression of opsin in developing photoreceptors and alters their number and morphology in mature retina. These alterations were accompanied by disorganization of inner nuclear and plexiform layers [27]. It may have a role in genetically predisposed thinned retina in RB patients in the present study.

It was suggested that viral oncoproteins may have a role in retinal development beside predisposition to tumorogenesis [28–31].

Another issue is the environmental signals to retinal cells by changing the intrinsic or extrinsic factors [32, 33].

Following monocular enucleation of RB patients in the present study, the contralateral clinically free eyes show thickened total macula significantly except in inferior sectors, the average GCL, GCC, and mean pRNFL as a whole and in quadrants of the optic nerve head except the nasal quadrant compared to same parameters in the contralateral clinically free eyes of RB patients not treated by enucleation. No statistically significant difference between study group (I) and the control group. Our finding here was that the thinning observed in study group (II) was overcome by thickening in study group (II) following enucleation to reach the normal thickness as in the control group. A statistically non significant difference between the study groups regarding the demographic and clinical data allowed us to do such a comparison.

The explanation of the plastic changes in the macula and optic nerve head of the remaining eyes after unilateral enucleation for RB was suggested to be actual increase in number of the ganglion cells and glial elements in an already thinned RB retina. A statistically significant increase in mean pRNFL in superior, temporal and inferior quadrants was detected due to extensive sprouting of nerve endings. We think that the increase in mean GCL and mean GCC were not significant because of associated decrease of the contralaterally projecting cells of the retina.

The net result between the actual increase in ipsilateral projection and the decrease in number of contralateral projecting cells in the retina was not significant. We couldn’t judge if there was a change in certain type of ganglion cells either in number and size, these need further histopathological studies. There was no explanation for more significant reduction in mean CFT after unilateral enucleation except that these increase in number of cells and glial elements involving more the inner retinal layers which are normally deficient in the fovea pit so the mean CFT here was still thin as found in study group (II).

These findings in macula and optic nerve head following unilateral enucleation in the present study can be explained by several animal studies [34–37]. They found a significant increase in the number of optic nerve axons occurs in dorsal lateral geniculate nucleus and in the superior colliculus on the side ipsilateral to the remaining eye [35, 37–39]. This apparent ipsilateral expansion may be explained in various ways; there might be no actual increase in the number of ipsilaterally projecting ganglion cells and the expansion may be due to a profuse sprouting of their axon terminals [36].

Another opinion was the rerouting of some retinal ganglion cell axons from the contralateral to the ipsilateral optic tract occurred without increase in ganglion cells number [36, 40] . Some authors suggested that neonatal monocular enucleation suppressed the otherwise occurring cell death of the ipsilaterally projecting ganglion cells [35] so that the latter appeared increased compared with those in normal rats [41].

More densely packed distribution of ganglion cells in lower temporal crescent region of the retina was found following monocular enucleation than in normal rats [42]. The second finding was expanded distribution of these cells in the upper hemiretina and lower nasal retina. This was in conjunction with their ealier results [43, 44]. A similar expansion of ipsilaterally projected ganglion cells into the upper nasal retina reported for retinocollicular pathway [39].

The third finding was decreased density of contralaterally projecting ganglion cells in retina compared with normal control. They found that some ganglion cells in the temporal retina have changed their axonal projection from the contralateral to the ipsilateral hemisphere and large to medium size cells lost in contralateral projection and gained in ipsilateral one [42]. This last finding about the type of ganglion cells involved in expansion was supported by Shirokawa et al. [45].

Lund et al. [46] proposed that some of the optic fibers which normally innervate the contralateral visual centers come to innervate the ipsilateral side, resulting in an increase in the uncrossed projections.

Ahmed et al. [47] proposed that the mechanism of persistence of bilaterally branched axons present early in the development. They found that initially the double-labeled cells are distributed over the entire retina but that over the course of development they are subsequently found in the ventral - temporal crescent in the normal adult albinos due to the withdrawal of axons belonging to retinal ganglion cells which die off.

One of the chemical factors which involved in neural plasticity after monocular deprivation is Retinal Brain Derived Neurotrophic Factor [48]. This may highlight the possibility of presence of chemical agents involved in the results of the effects of monocular enucleation on the contralateral eye which are not studied yet.

After unilateral enucleation in hamster at birth, examination of adult retinal ganglion cell layers (including retinal ganglion cells, displaced amacrine cells and glial cells [49]) in remaining eyes revealed increase by about 8%, with different distribution of cells according to cell size. The increase in cell number was found across the entire retina, but was largest in the temporal retina. [50]. The soma size is controlled by one factor which is the volume of axon of it had to maintain [39] also presence of overlap and competition increased terminal arborization and soma size [50].

There was a significant positive correlation between SAP pattern deviations and the pRNFL thickness in study group (I); increased thickness may be associated with further functional damage. So those survived RB children need a long term follow up by visual field for the remaining eyes. A study was conducted to evaluate the impact of RB on the health status of survivors in terms of disabilities and worries, both of which may restrict participation in activities of daily life. Fear of developing second primary tumors passing RB on to the next generation and of further loss of vision were important life-long problems they worried about [10].

## Conclusion

This study was the first one considering description of possible structural changes in the macula and optic nerve head of the clinically free eyes of RB survived patients. These eyes were characterized by thinning of CFT, GCL, GCC in the macula with no statistically significant difference in thickness of pRNFL compared to the control group. These changes may be due to the genetic defects in RB. After unilateral enucleation, increased thickness in macular GCC and pRNFL were detected compared to RB group and these changes were statistically correlated to SAP pattern deviations. This may highlight the importance of long term follow up examinations of those remaining eyes. Further histopathological studies are needed to confirm these findings.

## Abbreviations

Anova: One – way Analysis of variance
BCVA: Best corrected visual acuity
C/D: cup disc ratio
CFT: central foveal thickness
ETDRS: Early Treatment Diabetic Retinopathy Study
FMASU REC: Faculty of Medicine Ain Shams University Research Ethical Committee
GCC: ganglion cell complex
GCL: ganglion cell layer
INL: Inner nuclear layer
IOP: intraocular pressure
IPL: inner plexiform layer
IQR: interquartile range
Log.MAR: logarithm of minimum angle of resolution
mGCC: macular ganglion cell complex
mGCL: macular ganglion cell layer
mRNFL: macular RNFL
OCT: optical coherence tomography
ONL: outer nuclear layer
OPL: outer plexiform layer
pRNFL: peripapillary retinal nerve fiber layer
*P*-value: probability value
RB: retinoblastoma
RBp: retinoblastoma protein
RPE- IS/OS: Retinal pigment epithelium to /inner/outer segment junction of photoreceptors
SAP: standard automated perimetry
SD: Standard deviation
SPSS: statistical package for the social sciences
